# Hippocampal volumes are important predictors for memory function in elderly women

**DOI:** 10.1186/1471-2342-9-17

**Published:** 2009-08-22

**Authors:** Martin A Ystad, Astri J Lundervold, Eike Wehling, Thomas Espeseth, Helge Rootwelt, Lars Tjelta Westlye, Martin Andersson, Steinunn Adolfsdottir, Jonn Terje Geitung, Anders M Fjell, Ivar Reinvang, Arvid Lundervold

**Affiliations:** 1 Department of Biomedicine, Neuroinformatics and Image Analysis Laboratory, University of Bergen, Jonas Lies vei 91, 5009 Bergen, Norway; 2 Department of Biological and Medical Psychology, University of Bergen, Jonas Lies vei 91, 5009 Bergen, Norway; 3 Kavli's Dementia Research Centre, Haraldsplass Deaconess Hospital, 5009 Bergen, Norway; 4 Center for the Study of Human Cognition, University of Oslo, POB 1094, Blindern, 0317 Oslo, Norway; 5 Department of Medical Biochemistry, Rikshospitalet University Hospital, 0027 Oslo, Norway; 6 Department of Radiology, Haraldsplass Deaconess University Hospital, 5009 Bergen, Norway; 7 Department of Radiology, Haukeland University Hospital, 5021 Bergen, Norway

## Abstract

**Background:**

Normal aging involves a decline in cognitive function that has been shown to correlate with volumetric change in the hippocampus, and with genetic variability in the APOE-gene. In the present study we utilize 3D MR imaging, genetic analysis and assessment of verbal memory function to investigate relationships between these factors in a sample of 170 healthy volunteers (age range 46–77 years).

**Methods:**

Brain morphometric analysis was performed with the automated segmentation work-flow implemented in FreeSurfer. Genetic analysis of the APOE genotype was determined with polymerase chain reaction (PCR) on DNA from whole-blood. All individuals were subjected to extensive neuropsychological testing, including the California Verbal Learning Test-II (CVLT). To obtain robust and easily interpretable relationships between explanatory variables and verbal memory function we applied the recent method of conditional inference trees in addition to scatterplot matrices and simple pairwise linear least-squares regression analysis.

**Results:**

APOE genotype had no significant impact on the CVLT results (scores on long delay free recall, CVLT-LD) or the ICV-normalized hippocampal volumes. Hippocampal volumes were found to decrease with age and a right-larger-than-left hippocampal asymmetry was also found. These findings are in accordance with previous studies. CVLT-LD score was shown to correlate with hippocampal volume. Multivariate conditional inference analysis showed that gender and left hippocampal volume largely dominated predictive values for CVLT-LD scores in our sample. Left hippocampal volume dominated predictive values for females but not for males. APOE genotype did not alter the model significantly, and age was only partly influencing the results.

**Conclusion:**

Gender and left hippocampal volumes are main predictors for verbal memory function in normal aging. APOE genotype did not affect the results in any part of our analysis.

## Background

MR imaging and segmentation of brain volumes have been increasingly applied in studies of the human brain and its functions. Several studies on aging and age-related cognitive decline have combined neuropsychological tests with MRI findings. These studies have revealed a relationship between regional volumetric atrophy as measured with 3D MRI, decline in memory functions as measured with neuropsychological tests, and the presence of early signs of dementia [1-3].

Although environmental factors contribute to the variation in cognitive function during aging, recent studies have identified genetic markers as significant factors, not only for cognitive function, but also for volumetric variability in aging [4,5]. Studies on the genetic basis of cognitive decline and Alzheimer's Disease (AD) have pointed out Apolipoprotein E (APOE), a protein intimately involved in synaptogenesis but also numerous neuropathological processes [6], as an important marker. The risk of developing AD increases significantly by carrying one or more of the ϵ4-allele of APOE [7]. ApoEϵ4 is also associated with reduced memory function in patients with mild cognitive impairment (MCI) [8,9]. Smith and collaborators [10] studied a group of MCI patients diagnosed on the basis of memory deficits and found that ApoEϵ4 was associated with poorer performance on tests of learning and recall in MCI patients, but not in normal controls. They suggested that ApoE-related memory deficits are specific cognitive phenotypes in patients with AD pathology. In a group of non-demented older adults, Bondi and collaborators [11,12] found memory impairment at study entry in ApoEϵ4 carriers, affecting measures of recall, recognition discriminability, and learning as measured by the California Verbal Learning Test (CVLT).

Combining genetics with volumetric imaging has indicated that regional hippocampal volumes correlate negatively with the zygosity of ApoEϵ4 [13-16]. There are however conflicting results on this topic. Where some studies report no association between hippocampal volumes and APOE genotype [17-19], others have reported mainly longitudinal effects of ApoEϵ4 on hippocampal volumes [20-22], indicating that follow-up studies are more sensitive to pick up associations between APOE genotype and hippocampal volume, than are cross-sectional studies on healthy volunteers.

Furthermore, Tupler et al. [23] combined MRI-volumes of the hippocampus and APOE genotype in a five year follow-up study to investigate their relative contributions to cognitive decline, as measured by CVLT. The study concluded that, second to previous cognitive testing, ApoEϵ4 predicts memory decline in healthy controls and that MRI-morphometry of the hippocampus added only slightly to the predictive value. However, despite their important prospective design, some methodological weaknesses could be identified in this study. Firstly, the image segmentation and volumetric analysis were performed using manual ROI tracings and several technicians. This adds subjectivity as an error-source in the volumetric analysis. Secondly, the hippocampal volumes were adjusted for cerebral volume, age, sex, and the APOE age interaction. The former might cause a problem, as the cerebrum as a whole continually decreases in size during age [24,25], which makes it particularly inappropriate as a normalizing factor. Also, men and women have different volumetric fractions related to cerebrum size [26]. Another study conducted by Marquis et al. [27], concluded that previous cognitive test performance and hippocampal volume each predicted onset of questionable dementia, independent of age and sex, whereas possession of the ApoEϵ4 allele did not alter the prediction significantly.

In the present study of 170 normal elderly subjects, we set out to assess the relationship between (i) volumetry of brain structures involved in memory, (ii) genotype of the polymorphic ApoE gene and (iii) scores on the long delay free recall subtest from CVLT (CVLT-LD). Few studies other than Tupler et al. [23], Lind et al. [13], and Adak et al. [28] have combined all three measures to assess the combined predictive value of hippocampal volumes and APOE genotype on episodic memory measures in healthy subjects. Moreover, we have applied a recent multivariate statistical method of conditional inference to investigate the sequential importance of these two variables in predicting CVLT-LD. Our analysis also took into account the effect of age and gender in the analysis, investigating whether these variables could add to the abovementioned predictions.

Although we have performed only a cross-sectional study, in contrast to the repeated and predictive design by Tupler et al. [23], a major contribution besides conditional inference tree analysis, is the use of semi-automated image processing methods. This is designed to reduce subjectiveness in the analysis. Moreover, we use the total intracranial volume (ICV) as a normalizing factor, a measure which does not change with age. A major point of interest was also whether results from our two geographic groups; the Bergen sample and the Oslo sample, were similar and thereby justify pooling of the samples. We were using the same neuropsychological test procedures and independent use of the same brain segmentation-and volumetric software package, but with different MRI-scanners. This "two-center design" would reinforce the significance of any consistent findings and provide a strong background for further analysis using the methods described.

## Methods

### Subjects

The subject material consisted of 170 individuals (mean age 62.2 years; range 46–77; 120 females and 50 males). There were 86 (60 females) in the Bergen sample and 84 (60 females) in the Oslo sample. The participants were interviewed, and those with previous or present neurological or psychiatric disorders, head trauma, a history of substance abuse, or other significant medical conditions were excluded. Based on information from interviews and evaluation of neuropsychological test results, none of the subjects were defined as demented. All subjects signed an informed written consent to participate in the study, and the study was approved by the Regional Committee for Medical Research Ethics of Southern Norway. The age, education and IQ-range (summarized in Table 1) were largely similar in all age groups in both samples.

**Table 1:** Demographic characteristics of the study sample

Location	N	M	F	Age mean [yrs.] (range)	Education mean [yrs.] (range)	IQ mean (range)
Oslo	84	24	60	65.1 (47–75)	13.7 (9–19)	118.2 (86–145)
Bergen	86	26	60	59.3 (46–77)	13.9 (8–20)	115.5 (88–136)
Pooled	170	50	120	62.2 (46–77)	13.8 (8–20)	116.8 (86–145)

### MR image acquisition

**Figure 1:**
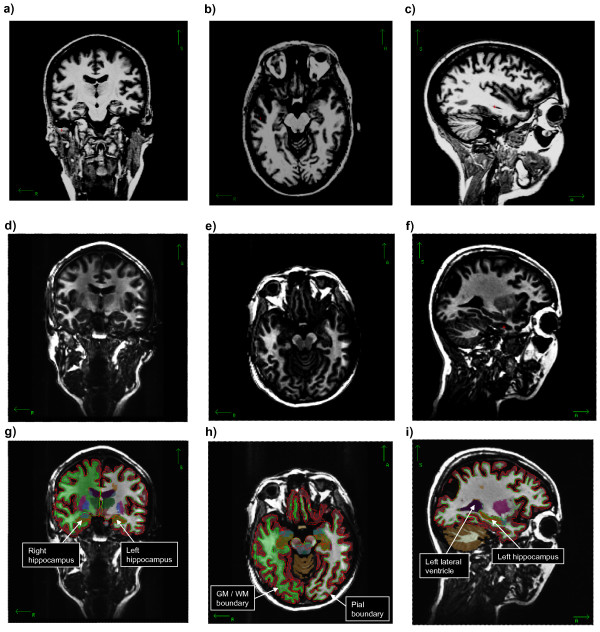
**Original MRI data (upper two rows) and color-coded segmented data (lower row)**. **a) **Coronal slice 120 from an Oslo dataset. **b) **Axial slice 120. **c) **Sagittal slice 160 transectioning left hemisphere. **d) **Coronal slice 120 from a Bergen dataset. **e) **Axial slice 112. **f) **Sagittal slice 160 transectioning left hemisphere. **g) **Same slice as in d) with pial boundary, gray matter/white matter boundary, and subcortical segmentation, with annotation of segmented left and right hippocampus. **h) **Same slice as in e) with overlayed segmentations and annotation of pial surface and gray matter/white matter boundary. **i) **Same slice as in f) with overlayed segmentations and annotation of segmented left lateral ventricle and left hippocampus.

A 3D T1-weighted MR-protocol suitable for clinical use was applied. The scans in Bergen were acquired on a 1.5 T GE Sigma Echospeed scanner with a standard 8-channel head coil, using 256 × 256 × 124 dual-volume sagittal T1-weighted 3D FSPGR IR prepared acquisitions (TR/TE/TI/FA = 9.5 ms/2.2 ms/450 ms/7°) at voxel-size 0.94 × 0.94 × 1.4 mm^3 ^(cf. Figures 1d–f). In Oslo, the volumes were recorded on a 1.5 T Siemens Sonata scanner with a conventional head coil, using 256 × 256 × 128 dual-volume sagittal T1-weighted 3D MPRAGE aqcuisitions (TR/TE/TI/FA = 2.730s/3.39 ms/1000 ms/7°) at voxel-size 1.0 × 1.0 × 1.3 mm^3 ^(cf. Figures 1a–c).

At both sites, the DICOM images were then transferred from the scanner to a Linux workstation for further processing as described below.

### MRI morphometric analysis

The volumetric analysis, using the FreeSurfer software package (ver. 3.03 in Oslo, and ver. 3.01 in Bergen) [29-39] was based on two consecutive T1-volumes acquired during a single examination. The volumes were averaged after coregistration to improve signal-to-noise ratio and obtain a better representation of the actual volume before skull stripping [29], normalization, and Talairach conversion was performed [30]. The automated procedures for volumetric measurements of the cortical mantle is described in [31-36], and the analysis techniques have been validated both histologically [37] and by manual measurement [38]. Furthermore, in a recent work reviewing several different automatic segmentation procedures [39], the current method obtained good results compared to other automated methods.

#### Surface and thickness measures

**Figure 2:**
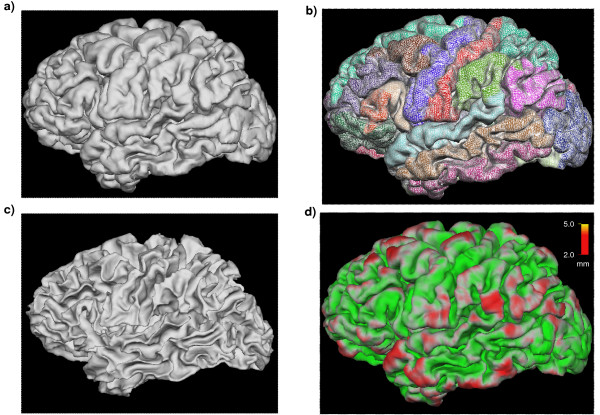
**Pial and white matter surface reconstructions**. **a) **Pial boundary. **b) **Pial boundary as wireframe surface with overlayed cortical parcellation map. **c) **White matter/gray matter boundary. **d) **Cortical thickness map derived from surfaces a) and c). The difference between the volume enclosed by the left and right pial surfaces and by the white matter/grey matter boundary surface is taken as the *total cortical volume*. Left and right hemisphere volumes in Table 3 are calculated as the intra-pial volumes.

Thickness measurements were obtained by reconstructing the gray/white matter boundary surface and the gray/pial boundary surface (Figures 1d–f and Figure 2) and calculating the distance between the two surfaces at each point in the cortical mantle. This method uses both intensity and continuity information from the entire three-dimensional MR volume in segmentation and deformation procedures to produce representations of cortical thickness. Thickness measurements are not dependent on the original resolution, and can therefore detect submillimeter differences between groups.

#### Subcortical volume measurements

Subcortical segmentation uses somewhat different methods than in the surface processing step, as described in [40] and [41]. This step of the segmentation produces volumes for a number of subcortical structures, including the hippocampus (cf. Figures 1d–f). Results from manual labeling of a training set, according to [42-44], are used to automatically label the subcortical structures. This procedure uses the information from the training set to determine the most probable tissue class for each voxel in the volume. This probability is based on the voxel's location in the volume, the neighboring voxels' tissue classes, and the intensity value in each voxel. This automatic labeling procedure has been shown to be comparable in accuracy to manual labeling [40].

#### Manual intervention on automatic labeling and normalization

Due to biological variability and differences in image quality, it was necessary to control the results after both the surface reconstruction and the subcortical segmentation processes. This had to be done for each subject. Errors in the surface reconstruction or subcortical labeling were corrected using the methods described in [41], and the appropriate part of the processing stream was re-processed. Average processing time for one subject was between 16–24 hours on a well-equipped Linux workstation. Manual intervention is a subjective procedure and the results differ slightly with different operators. Moreover, the quality of the T1-weighted acquisitions were slightly better in the Oslo sample than in the Bergen sample in that the latter sample suffered small magnetic field inhomogeneities. The algorithm for correction described in [41] was applied to both samples, with different operators in Oslo and Bergen.

By including 170 individuals of both genders and with different body size, one can expect a wide range of values in morphology and measures of body structures. In the brain, this can be appreciated by the fact that large human beings have larger somatosensory areas and more muscle to control and thus, larger brains. This is evident in the different brain sizes in men and women [26,45,46]. We used the ICV as a normalizing factor for structural brain sizes, as these ratios are not dependent on age, sex, or body size. Consequently, the ICV is constant throughout the adult lifespan, something that does not apply to Total Brain Volume (TBV), a measure that is used in other studies [25,45].

### Genetic analysis

The APOE genotype was determined with polymerase chain reaction (PCR) with allele-specific fluorescence energy transfer probes and melting curve analyses on the LightCycler system (Roche Diagnostics, Mannheim, Germany). As also explained in the study by Espeseth et al. [47], DNA was extracted form 300 *μ*L whole blood using MagNA PureLC DNA Isolation Kit-Large Volume on the MagNA Pure LC (Roche), eluted and diluted to 1 mL, of which 5 *μ*L was applied in each assay. The typing of ApoE-zygosity was performed using the LightCycler APOE Mutation Detection Kit (Roche). The assay was performed as specified by the supplier, except for scaling down the total assay volume from 20 to 10 *μ*L. The laboratory participates in an external quality assurance program (Equalis, Uppsala, Sweden) that includes APOE-genotyping. Individuals were characterized by their APOE allele combinations ϵ*i/j *(*i, j *= 2, 3, 4; *i *≤ *j*), and classified as ApoE-ϵ4-positive (ϵ4-carriers) or ApoE-ϵ4-negative (non-carriers) based on the presence or absence of at least one ϵ4 allele.

### Neuropsychological assessment

A Norwegian translation of the California Verbal Learning Test-II (CVLT) [48,49] was part of a neuropsychological evaluation. CVLT is a standardized test of verbal learning and memory function, developed to assess both the amount of material learned, recalled and recognized, as well as qualitative aspects of how the verbal learning occurs or fails. A list of 16 words (List A) is presented five times. The words are equally drawn from four semantic categories with no consecutive words from the same category. Immediately after the fifth trial, the participant is read a new list (List B) and asked to recall it. A short delayed recall test is presented immediately after recall of List B, where the participant is asked to recall the words in List A. A long delayed recall test (CVLT-LD) is presented after an interval of 20 minutes where the participant works with non-verbal tasks. Finally, a "yes-no" recognition test is presented, including the 16 items of list A, eight from list B, and 20 random distractor items. In the present study, we report one verbal learning measure (i.e. total learning across trial 1–5) and a delayed recall measure (i.e. CVLT-LD). The latter is used as a measure of verbal memory function in the conditional inference analysis. Two subsets (Vocabulary and Matrix Reasoning) from the Norwegian translation of the Wechsler Abbreviated Scale of Intelligence (WASI) [50] were used to estimate intellectual function (cf. IQ in Table 1).

### Statistical analysis

**Figure 3:**
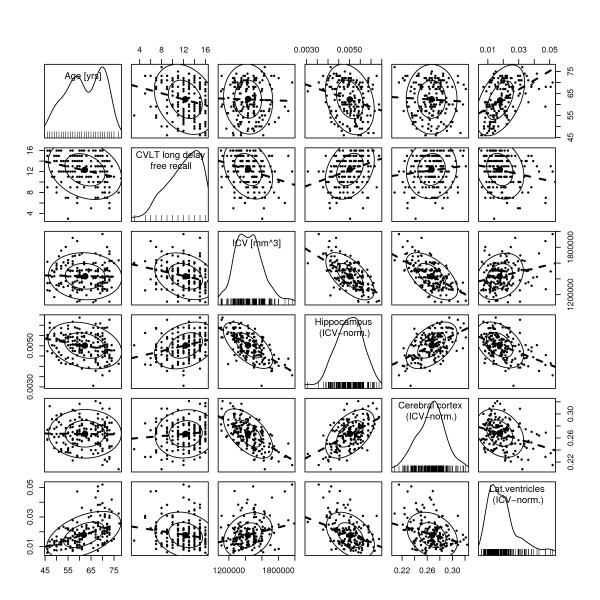
**Scatterplot matrix between all pairs of variables: age (years), CVLT free recall long delay (number of correct items), intracranial volume (ICV) in mm^3^, hippocampus volume (ICV-normalized), total volume of cerbral cortex (ICV-normalized), and total volume of lateral ventricles (ICV-normalized). **On the diagonal panels estimated probability density are given for each variable, together with measurement value for each observation, the latter in the form of vertical lines along the x-axis. The numerical range for each variable is given along both the horizontal and the vertical borders of the matrix plot. The off-diagnal panels allow for each variable to be compared to any other variable, interchanging the ordinate and the abscissa. For each of these bivariate scatterplots a least square linear regression line is fitted. Elliptic data-concentration contours for the fitted bivariate normal distribution are also plotted. For highly correlated data the elliptic shape is elongated, and for uncorrelated data the shape is circular. The contours are plotted at levels 0.5 and 0.9, i.e. 50% of the data is within the inner ellipse, and 90% within the outer one. (The figure was produced by the car package in R.)

**Figure 4:**
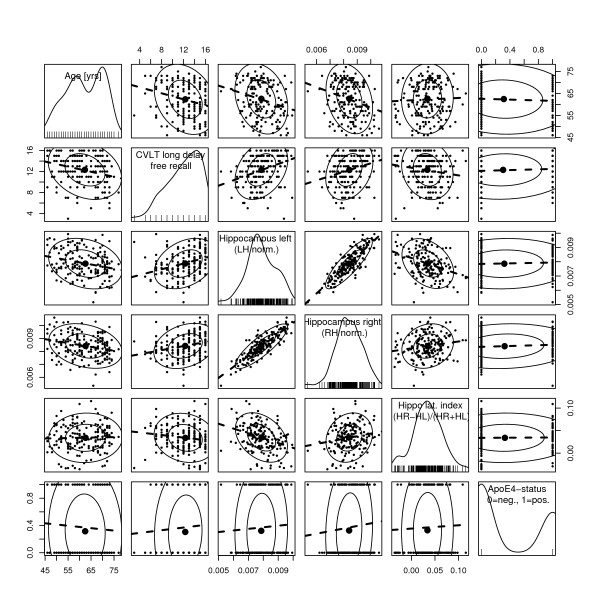
**Scatterplot matrix**. Age (years), CVLT free recall long delay (number of correct items), Left hippocampus volume (normalized to intra-pial volume of ipsilateral hemisphere), Right hippocampus volume (normalized to intra-pial volume of ipsilateral hemisphere), Hippocampal laterality index (Right hippocampus - Left hippocampus) = (Right hippocampus + Left hippocampus), ApoEϵ4 status (non-carrier = 0, carrier = 1). On the diagonal panels, the estimated probability density is plotted. Data-concentration ellipses are plotted in the off-diagonal panels at levels 0.5 and 0.9. For comprehensive explanation see Figure 3 legend.

**Figure 5:**
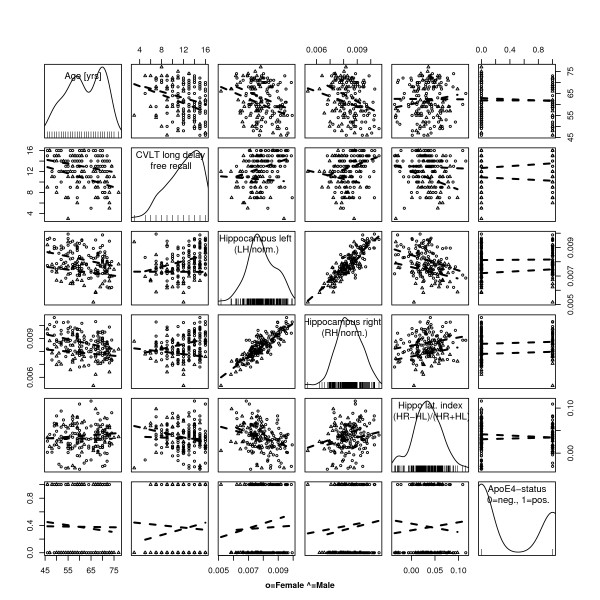
**Scatterplot matrix grouped by gender**. Age (years), CVLT free recall long delay (number of correct items), Left hippocampus volume (normalized to intra-pial volume of ipsilateral hemisphere), Right hippocampus volume (normalized to intra-pial volume of ipsilateral hemisphere), Hippocampal laterality index (Right hippocampus - Left hippocampus) = (Right hippocampus + Left hippocampus), ApoEϵ4 status (non-carrier = 0, carrier = 1). Notice the gender differences for CVLT performance versus hippocampal volumes. On the diagonal panels, the estimated probability density is plotted.

**Figure 6:**
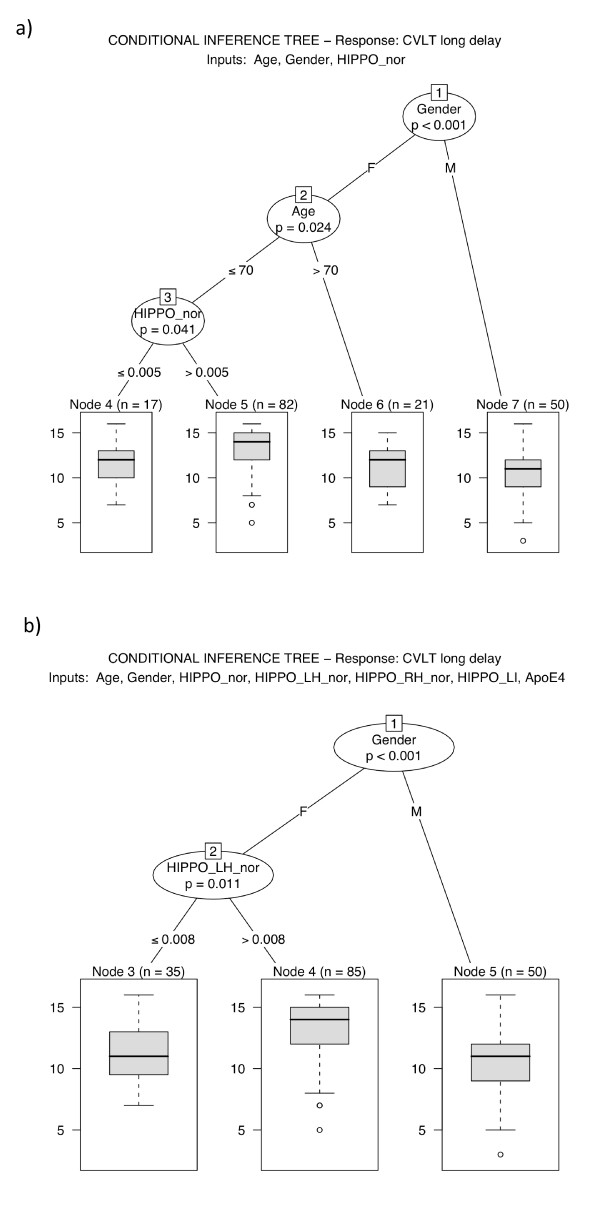
**Conditional interference tree *t *for predicting response *y *(CVLT free recall long delay) as a function of input variables *x***. **a) ***x *= {Age, Gender, ICV-normalized hippocampus volume (HIPPO_nor)}. **b) ***x *= {Age, Gender, ICV-normalized hippocampus volume (HIPPO_nor), Right hippocampus normalized by right (intra-pial) hemisphere (HIPPO_RH_nor), Left hippocampus normalized by left (intra-pial) hemisphere (HIPPO_LH_nor), Hippocampal laterality index (HIPPO_LI), ApoEϵ4 status}.

For descriptive analysis we used matrix scatterplots and simple linear regression models with estimation and plotting of the probability distribution of each variable (Figures 3, 4 and 5). The MATLAB^® ^scientific programming package was used for this analysis and graphics. To assess possible complex relationships between morphometric, behavioural, and genetic variables we used the novel technique of conditional inference trees as implemented in R (version 2.7.0) software environment for statistical computations and graphics [51]. This particular kind of recursive conditional inference takes into account the distributional properties of the measures. Severeal covariates are included in the model, and one response variable is defined. The conditional inference model states, that if the null-hypothesis of there being independence between any of the covariates and the response cannot be rejected, the variable in question is excluded from further exploration. However, when one variable distinguishes itself by having the strongest association with the response, a split is created with two disjoint sets of the variable in question. The Bonferroni adjusted p-value of the split value is calculated. For each such node, the abovementioned procedure is repeated for each condition until none of the covariates can reject the null-hypothesis. From this data we calculated a statistical decision tree as shown in Figures 6a and 6b.

## Results and discussion

### Results

#### Genetic analysis

The ApoEϵ4 carrier group comprised 63 subjects (37% of the study group), with allele combinations ϵ2/4 (*n *= 7), ϵ3/4 (*n *= 48) and ϵ4/4 (*n *= 8). Thus, 4.7% of the sample was ϵ4/4 homozygote. In the non-carrier group of 107 subjects the allele combinations were ϵ2/3 (*n *= 21) or ϵ3/3 (*n *= 86). No subject had the allele combination ϵ2/2. Wilcoxon rank sum test for equal medians revealed no statistically significant difference for CVLT-LD scores between subjects with two, one, or zero ApoEϵ4 alleles at 5% significance level. Using the same test to compare homozygote ϵ4ϵ4 ApoE-carriers (n = 8) to the rest of the sample did not reveal any significant difference, neither in normalized hippocampus volume, nor in CVLT-LD. Median values for normalized hippocampal volumes were 0.0049 (range 0.0031–0.0061) and 0.005 (range 0.0036–0.0064), and CVLT-LD scores were 12 (range 7–16) and 13 (range 3–16) for ApoEϵ4ϵ4 versus others, respectively.

#### Brain morphometry

Main morphometric results by gender and study location are presented in Table 2. There were no statistically significant difference in mean values between the Oslo and the Bergen sample for any of the brain structures being analyzed.

**Table 2:** Volumetric data from Oslo and Bergen samples, separately.

Location	Gender	ICV	TBV	LHvol	RHvol	Left Hippo. Vol.	Right Hippo. Vol.
Oslo	Male	1592.2 (155.7)	1089.3 (82.7)	489.7 (40.1)	488.3 (38.0)	3.40 (0.39)	3.67 (0.39)
	Female	1404.2 (103.4)	976.4 (74.5)	436.4 (33.5)	436.4 (32.9)	3.36 (0.31)	3.61 (0.36)

Bergen	Male	1579.7 (165.3)	1147.1 (116.0)	472.2 (44.7)	475.0 (42.2)	3.59 (0.44)	3.86 (0.44)
	Female	1358.3 (128.7)	1016.2 (89.2)	411.7 (36.6)	413.4 (36.2)	3.51 (0.34)	3.71 (0.36)

Pooled	Male	1585.7 (159.3)	1119.4 (104.6)	480.6 (43.0)	481.4 (40.4)	3.50 (0.42)	3.77 (0.43)
	Female	1381.3 (118.5)	996.3 (84.2)	424.0 (37.0)	424.9 (36.4)	3.43 (0.33)	3.66 (0.36)

Volumes are given in mL as mean and standard deviation. ICV = intracranial volume, TBV = total brain volume, LHvol = left hemisphere volume, RHvol = right hemisphere volume, Hippo. Vol. = hippocampus volume.

Scatterplot matrix with pairwise comparisons of the different variables together with corresponding linear regression lines are presented in Figures 3, 4 and 5. A linear least-squares regression analysis supports the notion that the hippocampus, as the rest of the brain, shrinks with age (0.03 mL/year for men, and 0.029 mL/year for women). Another finding was that the hippocampus volume declines relatively more than the cortical volume with age, adding to the significance of this particular structure in age-related substance loss. On the other side, the relative size of the lateral ventricles increase with age (0.89 mL/year for men, and 0.77 mL/year for women). The size of the lateral ventricles is a commonly used marker for age related substance loss in neuroradiological practice.

Lateralized hippocampal analysis (Figure 4) revealed that the right hippocampus is on average slightly larger than the left across all age groups (right hippocampus volume: mean (SD) = 3.693 (0.383) mL; left hippocampus volume: 3.452 (0.362) mL; paired t-test: p < 0.00001, 95% confidence interval for the mean difference: 0.209–0.274 mL), also when normalized by their respective ipsi-hemispherical volumes (p < 0.00001). Scatterplot matrix of the variables age, CVLT-LD, hippocampal volumes, and ApoEϵ4-status grouped by gender is presented in Figure 5, showing that the left and right normalized hippocampal volumes decline with age for both men and women, and that the bilateral normalized hippocampus volume is slightly larger for women than for men.

#### Verbal memory function

CVLT-scores by sample and gender are presented in Table 3. The CVLT scores produced a negatively skewed distribution in our sample (Figures 3, 4 and 5). In our study there was an age related decline in the long delay subtest of the CVLT (CVLT-LD) in both males and females. Also, women outperformed men on average CVLT-scores (p < 0.0001) (Figure 5). Linear least-squares regression analysis showed a small but statistically significant positive relation between CVLT-scores and total hippocampus volumes (R^2 ^= 0.08, p < 0.0001).

**Table 3:** CVLT scores from Oslo and Bergen samples, separately

Location	Gender	Total Learning mean (sd)	CVLT Long Delay mean (sd)
Oslo	Male	46.7 (11.9)	11.0 (3.0)
	Female	52.5 (9.4)	12.4 (2.7)

Bergen	Male	45.3 (8.6)	10.3 (3.1)
	Female	55.4 (8.7)	13.1 (2.6)
Pooled	Both	51.6 (10.2)	12.1 (2.9)

#### Conditional inference

When including age, gender and normalized hippocampal volume as explanatory variables and CVLT-LD as response variable, we calculated the conditional inference tree as depicted in Figure 6a. Gender is shown to be the most influencial variable (Bonferroni adjusted p < 0.001). This variable followed through for men, leaving it to predict CVLT-LD for this part of the sample without any other variables adding to the predictive value. For women, both age and hippocampal volumes produced statistically significant splits in the sample regarding CVLT-LD scores (Figure 6a) (Bonferroni adjusted p = 0.024 and 0.041, respectively).

For women under 70 years, which constituted the majority of our sample, the bilateral hippocampal volumes created a split at normalized volumes below and above 0.005. Women less than 70 years who had normalized volume below 0.005 (n = 17) performed significantly poorer on the verbal memory score than those with a higher volume (n = 82).

When we included additional morphological variables related to left and right hippocampus, as well as ApoEϵ4 status to the set of explanatory variables, we obtained an even simpler regression tree result. As expected, gender was still the most important variable, and males were separated from the sample and no other variables could reject the null-hypothesis for men, i.e. explain the variability in CVLT-LD score. However, age for women now vanished as a predictor, leaving only the *left *normalized hippocampus volume as the more powerful predictor, creating a split at normalized volume of 0.008 (Bonferroni adjusted p = 0.011). Moreover, ApoEϵ4 status did not provide any useful predictive value in this sample, as was expected due to the findings mentioned above. The other hippocampus-related parameters were also shown to be little influential in prediction of verbal memory scores.

## Discussion

The present study is one of few studies of normal aging where image-derived hippocampal volumes, APOE genotype, and verbal memory performance have been jointly investigated. In our investigation we also included other variables (e.g. age, gender, hippocampus laterality index) that could be important a priori for prediction of verbal memory performance (i.e. CVLT-LD score). Applying the conditional inference model to our multivariate sample, we expected to reveal the sequential importance for each variable in predicting CVLT-LD.

Investigating our results (Figure 6a) we see that gender is the one variable that best predicts CVLT-LD in our sample. This is in concordance with findings in other studies of verbal memory function among elderly normals [52,53], where women in general, outperform men [1,52]. For men, there were no other variables that could be used to reject the null-hypothesis of there being no relationship between variable and response. For women however, age provided another node at 70 years, inferring that women over 70 years perform poorer than those at 70 years or less. The lack of predictive variables in men might indicate a power-problem reflecting on the small number of male participants. However, one could also argue that the findings reflect the role of hippocampus in verbal memory. We found a slightly positive relationship between hippocampal volume and CVLT-score, which is in concordance with Walhovd et al., who found a correlation between CVLT free recall after 11 weeks, but not after 5 minutes [54]. As also found, the size of the hippocampus declines by about 0.03 mL/year in both men and women, and given the smaller overall hippocampal volume in women, this would indicate a more prudent role for hippocampal volume in verbal memory in women.

We also found that APOE genotype did not have any important predictive value regarding CVLT-LD score. This was also confirmed by the more simple Wilcoxon rank sum test. This result is in accordance with Smith et al. [10], who reported that the phenotypical significance of ApoEϵ4 seems to apply only in patients with diagnosed MCI or AD, but not in healthy controls [55,56]. However, others [11,12] have reported lower CVLT-LD scores in healthy elderly ApoEϵ4-positive individuals, suggesting that ApoEϵ4-related memory changes precede a clinical MCI or AD diagnosis.

The role of left hippocampal volume in verbal memory function has been reported in several previous studies [57-61], but the exact contribution of this structure in relation to ApoEϵ4-zygosity is still unclear. Hippocampal volumes have, in most cases, been reported second to ApoEϵ4-zygosity in importance at follow-up, or of no importance at all at baseline measurement [23]. It is remarkable that in our sample, the role of ApoEϵ4-zygosity, but also age, is negligible compared to that of left hippocampal volume in predicting verbal memory performance. This suggests a prominent role for the left hippocampal volume in the hierarchy of predictors to verbal memory function. Furthermore, since our analysis did not reveal any significant associations between hippocampal volumes and ApoEϵ4-zygosity, our investigation does not support the notion that changes in hippocampal volumes are, in fact, results of APOE genotype. The lack of findings relating to the APOE genotype may reflect our sample, consisting of healthy, rather well-functioning men and women. If one were to expand the "normality" criteria to include more subjects, one might find stronger APOE genotype correlations.

One other finding was that the hippocampus volume declines relatively more than the cortical volume with age (Figure 3). This is contradictory to the common perception that the cortex is more prone to age related change than the hippocampus. However, due to the high age distribution in our material, we postulate that these findings reflect an accelerated hippocampal atrophy occuring in advanced age [62,63]. A weakness in our study is the small number of participants. A total of 170 subjects could be too low, and only make for weaker statistical inference than would a larger sample. The sample selected for this study was also found to be rather homogeneous, something which is reflected in the above-normal distribution of education, IQ, and the age range (Table 1). However, in the literature there are indications that the effects of several predictive factors are only surfacing in the older segment of the population [36,64], thus questioning the significance of including young people in studies of age-related disease. When comparing the results to other aging studies one should be cautious, considering the slightly younger age group in our sample. This age composition produces a smaller variance in cognitive function as compared to other aging studies. However, the selection criteria were motivated by the desire to include only healthy elderly people, and to allow for follow-up studies of the same participants, where cognitive decline, neurodegeneration, and morphological abnormalities are expected to occur on a larger scale.

A possible confounder in our study is the different scanners used to acquire the brain volumes. This is however, a substantial difficulty in multi-center studies, as various scanner vendors provide a wide range of models with different specifications such as field strength, gradient system, coils, and pulse sequence principles and parameters. This might not present as a problem in clinical practice, or when manual deliniations and segmentations are performed, whereas automated methods can be more sensitive to subtle differences in image properties [39]. However, this apparent weakness in the study can also be considered as a strength, as it demonstrates the methodological robustness behind the results. The fact that we obtained similar findings in two independent samples (i.e the Oslo and the Bergen material), also in terms of e.g. hippocampal volumes and its lateralization, is a significant strength of our study.

The automated image segmentation method used is well proven, and correlates well with more conventional, but time-demanding and subjectivity-prone methods [37,38]. This particular set of algorithms also produce reliable results compared to other, freely available software packages [39]. Multivariate methods based on conditional inference trees were applied in our analysis. These are rather novel methods in the applied statistical community [51,65], and have to a very little extent been used or known to the medical imaging and aging research community. However, such types of classification and regression trees (CART), e.g. [66], can provide robust and easily interpretable results.

A follow up study will be required to further investigate our findings. Such a study will provide an opportunity to do follow-up analysis much like Tupler et al. [23], and thereby make stronger inferences concerning predictive values from our variables. Furthermore, one would benefit from methodological improvements in magnetic resonance imaging and data-analysis made in recent years. It would therefore be interesting (and feasible) to acquire data with different MRI measurement techniques during the same imaging session, such as diffusion tensor imaging (DTI) and functional MRI (fMRI). Diffusion tensor imaging has given valuable biological information regarding 'white matter integrity' in age-related cognitive decline [67,68]. In addition, a particular kind of BOLD fMRI examination, called resting-state fMRI (RS-fMRI) or task-free fMRI, has shown to be sensitive to neuropsychological/neurodegenerative diseases, such as AD, in that appropriate analysis of such data can reveal a disruption in functional resting-networks in the brain. The RS-fMRI method has a genuine potential as a biomarker of disease and also as an early objective marker of treatment response, but needs to be further investigated [69,70].

## Conclusion

Using automated brain morphometric analysis, APOE genotyping, assessment of verbal memory function, and multivariate statistical methods based on conditional inference trees in a sample of 170 subjects (age range 46–77 years), we found that gender and left hippocampal volumes are main predictors for verbal memory function in normal aging. APOE genotype seems not to have any significant effect on verbal memory function. Reduced left side hippocampus volume seems more important than right side reduction for verbal memory decline in women, especially in women 70 years of age or less. Moreover, men seem to have more affected verbal memory function in this age group than women, and this memory decline in men seems not to be related to hippocampal volume. To further investigate these findings, we are about to conduct a follow-up study, using the same data collection protocols in both Bergen and Oslo.

## Competing interests

The authors declare that they have no competing interests.

## Authors' contributions

MY was involved in data preparation, morphometric analysis, statistical analysis, main author. AJL was involved in study design, supervisor, internal review. EW was involved in data collection, neuropsychological assessment, internal review. TE was involved in sata collection, neuropsychological assessment, internal review. HR was involved in APOE-genotyping, internal review. LTW was involved in data preparation, morphometric analysis, internal review. MA was involved in data collection, neuropsychological assessment, internal review. SA was involved in data processing, internal review. JTG was involved in data acquisition, internal review. AMF was involved in data processing, internal review. IR was the principal investigator of the main study in Oslo and Bergen, and participated in study design, internal review. AL was the project supervisor, and participated in MRI acquisition protocol design, statistical analysis, internal review.

## Pre-publication history

The pre-publication history for this paper can be accessed here:

http://www.biomedcentral.com/1471-2342/9/17/prepub
